# A description of health care system factors in the implementation of universal weight management services for children with overweight or obesity: case studies from Queensland and New South Wales, Australia

**DOI:** 10.1186/s13012-018-0801-2

**Published:** 2018-08-07

**Authors:** Helen A. Vidgen, Penelope V. Love, Sonia E. Wutzke, Lynne A. Daniels, Chris E. Rissel, Christine Innes-Hughes, Louise A. Baur

**Affiliations:** 1 School of Exercise and Nutrition Sciences, Faculty of Health, Queensland University of Technology, Victoria Park Rd, Kelvin Grove, Brisbane, 4059 QLD Australia; 2 Institute of Physical Activity and Nutrition, Faculty of Health, Deakin University, Locked Bag 20001, Geelong, 3220 VIC Australia; 3 The Australian Prevention Partnership Centre, PO Box K617, Haymarket, Sydney, 1240 NSW Australia; 4 NSW Office of Preventive Health, Locked Bag 7103, Liverpool BC, 1871 NSW Australia; 5 Discipline of Child and Adolescent Health, Clinical School, The Children’s Hospital, University of Sydney, Locked Bag 4001, Westmead, 2145 NSW Australia

**Keywords:** Child health, Universal care, Upscale, Obesity management, Weight management, Implementation, Health service

## Abstract

**Background:**

The prevalence of childhood obesity poses an urgent global challenge. The World Health Organization (WHO) Commission on Ending Childhood Obesity recommends the provision of appropriate family-based, lifestyle weight management services through universal health care to support families of children with overweight or obesity; however, there are few examples of their implementation ‘at scale’. The purpose of this research was to compare and contrast the impact of system and organisational factors on the implementation of childhood obesity management services within two Australian States (New South Wales and Queensland) to comprehensively describe their influence on the achievement of the WHO recommendation.

**Methods:**

Purposeful stratified sampling was used to select health service study sites (*n* = 16) representative of program implementation (none, discontinued, repeated) and geographic location within each State. Within each health service site, staff involved in program delivery, co-ordination and management roles participated (*n* = 39). An additional 11 staff involved in implementation at State level also participated. The Consolidated Framework for Implementation Research (CFIR) was used to develop interview scripts. Telephone interviews were recorded and transcribed. Transcripts were thematically coded and scored according to CFIR constructs and rating rules to identify enablers and barriers to implementation according to sample characteristics.

**Results:**

New South Wales achieved ongoing implementation; Queensland did not. Enablers included a quality evidence-based program, State government recognition of the urgency of the health issue and a commitment to address it, formally appointed and funded internal implementation leaders, strong communication and reporting at all levels. Barriers included the complexity of the health issue, in particular a lack of clear roles and responsibilities for local health service delivery, inadequate ongoing funding and challenges in meeting the diverse needs of families.

**Conclusions:**

This research is an important progression of the evidence base in relation to the translation of childhood obesity management trials into routine health service delivery. Understanding enablers and barriers to program implementation ‘at scale’ is imperative to inform future planning and investment by Australia and WHO member states to meet their commitment to deliver childhood weight management services as part of universal health coverage.

**Electronic supplementary material:**

The online version of this article (10.1186/s13012-018-0801-2) contains supplementary material, which is available to authorized users.

## Background

The prevalence of childhood obesity poses an urgent global challenge, with an estimated 41 million children under 5 years old being overweight or obese [1]. In Australia, overweight and obesity now affect 1 in 4 children aged 5–17 years (20.2% overweight; 7.4% obesity) [2]. Further, childhood overweight and obesity tracks into adulthood, with associated chronic disease [3, 4].

The World Health Organization (WHO) report of the Commission on Ending Childhood Obesity [5] urges the implementation of an integrated package of recommendations across the life course to provide prevention and management services. The provision of appropriate family-based, multi-component, lifestyle weight management services through universal health care is one of six evidence-informed recommendations from the WHO to support families of children who are already overweight or obese.

The evidence base of clinically trialled programs for children with overweight or obesity is strongest for the 5–12-year age group; however, current implementation approaches are resource-intensive and unsustainable in the long term [6]. The implementation of programs ‘at scale’ in a sustained manner presents a major challenge to the health sector if they are to respond adequately to the need for the provision of childhood obesity management services through universal health care.

### The Australian health care system context

Australia has no universal public health service available to families of children with overweight or obesity, no routine monitoring or screening of growth and weight status in children and no national health coverage to facilitate services through the primary health care system in the absence of comorbidities.

In Australia, National, State and Local governments share the responsibility for the delivery of prevention services and child health primary care. This can result in ambiguity and, therefore, inconsistency in service availability. In addition, the management of overweight and obesity can be viewed as a paediatric clinical service or the prevention of adult chronic disease, making its position in the health care continuum variable. This can result in a lack of clarity regarding responsibility for service delivery. Consequently, across most Australian States and Territories, the provision of childhood weight management services through public health services is ad hoc and inconsistent.

In 2008, funding in the form of the National Partnership Agreement on Preventative Health (NPAPH) was provided to all Australian States and Territories to address the rising prevalence of lifestyle-related chronic disease over an intended 7-year period [7]. The provision of evidence-based childhood obesity prevention and management programs across the life course was identified within State and Territory NPAPH Healthy Children Initiative implementation plans, with two of the States, Queensland (QLD) and New South Wales (NSW), delivering the PEACH™QLD and Go4Fun® programs, respectively.

The outcomes of PEACH™ [8, 9] and Go4Fun® [10, 11] demonstrate that these childhood weight management programs offer small but significant health benefits and are a much needed supplementary intervention to health service provision. An intervention is only beneficial, however, if implemented and maintained over time [12]. Understanding factors that influence the uptake and ongoing implementation of interventions is therefore emerging as an important consideration, alongside reach and impact, in determining the long-term viability of intervention efforts, the efficient allocation of resources and the translation of the intervention into other contexts. Such evidence can enhance the embedding of interventions into routine practice, dissemination into other settings and protection from adverse changes to funding and policy [13]. This is particularly important with regard to childhood weight management interventions given the prevalence of childhood obesity and its contribution to lifelong health.

The purpose of this research was to describe the enablers and barriers to ‘at scale’ implementation of childhood obesity management programs using PEACH™QLD in Queensland and Go4Fun® in NSW as case studies. ‘At scale implementation’ was defined as ‘deliberate efforts to increase the impact of successfully tested health interventions so as to benefit more people and to foster policy and program development on a lasting basis’ [13]. Categories of implementation were defined as program delivery having never occurred, having discontinued or having repeated implementation since Statewide commencement of the respective programs in NSW (2011) and in Queensland (2013). In NSW, discontinued sites have ongoing availability to the program should it become viable in their area. In this regard, they could be considered to have suspended implementation.

Constructs are described at two levels: the programmatic level, investigating factors affecting program implementation from the perspective of local health service sites within and between each State, and the systemic level, investigating factors affecting program implementation from the perspectives of central agencies between each State, and in comparison with their respective local health service sites. While programmatic level findings will inform strategies to address local implementation challenges, exploring systemic factors is likely to provide greater insight into the environmental, organisational and policy level strategies required to embed implementation into ongoing routine health service delivery.

More specifically, this research asks:

- To what extent were overarching corporate systems important to ongoing service delivery, i.e. were the key barriers and enablers for ongoing service delivery consistent across States for sites with similar levels of implementation?
- Within each State, did the perspectives of central agencies reflect the barriers and enablers identified by service sites? Did this vary according to level of implementation?

This research was conducted during a period when both Queensland and NSW Health Departments were funding the implementation of their respective programs. In 2017, the Queensland Department of Health ceased funding and co-ordinating PEACH™QLD centrally, with all local service providers choosing to discontinue the program as a result of having no central agency support system. The NSW Health Department continues to fund and co-ordinate Go4Fun®. This research, therefore, describes the impact of system and organisational factors on implementation by contrasting two States who differed in this way. The impact of these factors was examined at multiple layers within each State to comprehensively describe their influence on the achievement of the WHO Commission on Ending Childhood Obesity goal of embedding childhood weight management services into universal service delivery. At a State level, NSW has achieved this but Queensland has not and could be described as unsustained. However, during the active funding period of PEACH™QLD, when this data was collected, health service sites differed in their level of implementation. As a result, a final research question asks:

- Given that NSW achieved ongoing service delivery and Queensland did not, was there a difference in the perceptions of central agency staff between States?

## Methods

### Case studies and context

The need for this exploratory research emerged during the implementation of PEACH™QLD. The team at QUT were concerned about the sustainability of the project beyond the funding period. While they had considered this at the commencement of PEACH™QLD, strategies they had used appeared to not adequately identify key enablers for ongoing sustained service delivery [14]. Their neighbouring State, NSW, had achieved this with the Go4Fun®. An initial meeting of the implementation and funding teams from both States confirmed that the programs were comparable in aspects of program development, design, content and evaluation outcomes (Table 1); however, the implementation context for each program differed significantly. In NSW, program implementation was centrally co-ordinated within the State Health Department, supported by annualised funding with incentivisation to regional health services for program delivery. In Queensland, the State Health Department outsourced program co-ordination and implementation for a 3-year trial period, with local program delivery by regional health services being voluntary.

**Table 1:** A comparative summary of the PEACH™QLD and Go4Fun® programs

Program	PEACH™QLD	Go4Fun®
Evidence base	PEACH™/HELPP [8, 9]	UK MEND [34, 35]
Developer in Australia	Flinders University	Better Health Company
Funder	QLD Department of Health	NSW Ministry of Health
Commencement in Australia	2013	2011 (phased scale-up from 2009)
Eligibility criteria	5–11-year-old (primary school aged) children above the healthy weight range for age (weight category removed in 2016); parent/carer available to attend each session	7–13-year-old (primary school aged) children above the healthy weight range for age; parent/carer available to attend each session
Cost to participant	Free	Free
Venues	Community/school and health services	Community-based settings
Format and content	1.5 h face-to-face—healthy lifestyle changes through the development of parenting skills (parents only) and physical activity (child only); online option (2016)	2 h face-to-face—1-h nutrition (parent/carer and child) + 1 h game-based physical activity (child)/discussion on facilitated behaviour change (parent/carer) Online option (in development)
Frequency and timing	1.5 h/week (15 h total) After school hours, during school term	2 h/week (since 2014—previously 4 h/week) (20 h total) After school hours, during school term
Duration	6-month program of 9 weekly meetings with 10th meeting at 6 months post commencement; individualised family support by phone and text message between sessions 9 and 10	10-week program of 10 weekly meetings (since 2014) (prior: twice weekly meetings)
Follow-up	Family handbook; website; Facebook page	Access to Active 8 Website; sent a quarterly newsletter for 12 months following completion
Advertising and recruitment	QUT—website; Facebook; media; partnerships (NGOs, local government, health and non-health services); health professionals; local media; community groups Self-referral—via website or toll-free number	NSW health—website, Facebook Health services sites—local media, school newsletters, partnerships (NGOs, school nurses, health services, youth clubs, GPs) Self-referral—via toll-free number
State-wide co-ordination	QUT—project manager	NSW Office of Preventive Health—State program manager
State-wide monitoring	Flinders University	NSW Office of Preventive Health
State-wide training and support	QUT and Flinders University 2-day facilitator training Ongoing access to program and evaluation support Contact made at the commencement and completion of each program	Better Health Company and NSW Office of Preventive Health 2-day face-to-face training for program managers and leaders Annual professional development day for program managers Professional development via webinars for leaders Regular support teleconferences
Local co-ordination	Various (QUT, health services, tertiary institutions)	Health services through health promotion services
Local delivery	Trained facilitators	Trained leaders
State-wide evaluation	Flinders University	NSW Office of Preventive Health research in partnership with University of Sydney [10, 11] and Better Health Company
Program effectiveness	⇑ Fruit intakes ⇑ Vegetable intakes ⇑ Physical activity ⇓ Screen time ⇓ BMI *Z*-score	⇑ Fruit intakes ⇑ Vegetable intakes Decreases in sugar sweetened beverages ⇑ Physical activity ⇓ Screen time ⇓ BMI *Z*-score
Program reach	1122 children + 380 (online) (July 2013–June 2016) 501 children/annum	7821 children (July 2011–June 2016) 1564 children/annum
State prevalence: overweight and Obesity (2–17 years) [2]	24.6	24.8
State prevalence: overweight (2–17 years) [2]	17.9	16.9
State prevalence: obesity (2–17 years) [2]	7.5	8.7

upward arrow (⇑) denotes an increase

downward arrow (⇓) denotes a decrease

Both implementation teams included former and current central office staff of State Health departments who understood the contribution that clarity over elements which contributed to the effectiveness of these broader systems could have on securing funding for ongoing service provision for families. Despite the high global prevalence of this health issue, there are few published papers which describe the implementation of childhood obesity management programs ‘at scale’ [10, 15, 16]. There was an interest in exploring the breadth of implementation elements. The research design also sought to explore if there was consistency in elements which those designing program implementation (i.e. in central agencies) considered critical and the perspectives of those involved in service provision (i.e. health service sites).

### Theoretical framework

A range of implementation models were reviewed and the Consolidated Framework for Implementation Research (CFIR) [17, 18] chosen because it offered the opportunity to look broadly at possible key enablers and barriers to implementation but with sufficient specificity to direct future action and investment. The CFIR comprises 37 constructs across 5 domains, each of which is considered important for the implementation of innovations as routine practice (Table 2). While focusing on factors influencing implementation, the CFIR constructs have strong overlap with emerging contemporary sustainability constructs, such as strategic planning and evaluation; program adaptation and evolution; building organisational and community capacity; ensuring a supportive context; effective partnerships, commitment and support; and funding stability [12, 19]. The application of the CFIR constructs is context specific and therefore not all constructs need be examined. This study chose to use all, but one CFIR construct, given the paucity of weight management research available in which the CFIR has been applied. Trialability was the only CFIR construct to be excluded as both interventions in this study had undergone clinical trials. In addition, while each State had their own processes to assess program implementation, this research was more specifically interested in the influence of upstream organisational and system factors.

**Table 2:** Consolidated Framework for Implementation Research Constructs as examined by this study (adapted from Damschroder et al.) [17]

Domain	Construct
CFIR #1: Characteristics of intervention	#1.1 *Intervention source* development and implementation decision-making process #1.2 *Strength and quality of evidence* to support choice of intervention #1.3 *Relative advantage* of implementing intervention versus an alternative #1.4 *Adaptability* of intervention to meet local needs #1.5 *Trialability* of intervention prior to implementation #1.6 *Complexity* and difficulty of implementation #1.7 *Design quality and packaging* of intervention #1.8 *Costs* associated with implementation
CFR #2: Outer setting (external organisational environment)	#2.1 *Patient needs and resources* met in relation to implementation barriers/enablers #2.2 *Cosmopolitanism* (organisation networks with other external organisations) #2.3 *Peer pressure* to implement intervention #2.4 *External policy and incentives* (mandates, strategies) to spread intervention uptake
CFIR #3: Inner setting (internal organisational environment)	#3.1 *Structural characteristics* of the organisation, such as maturity, age and size #3.2 *Networks and communications* (informal or formal) within organisation #3.3 *Culture*, norms, values and basic assumptions of the organisation #3.4 *Implementation climate* (receptivity, compatibility, relative priority, incentives for change) #3.5 *Readiness for implementation* (leadership engagement and commitment, available resources, access to knowledge, information incorporated into work tasks)
CFIR #4: Characteristics of program implementers (facilitators)	#4.1 *Knowledge and beliefs about the intervention* and value placed on intervention #4.2 *Self-efficacy*/belief in own capabilities to implement intervention to achieve goals #4.3 *Individual stage of change* (level of preparedness to implement intervention) #4.4 *Individual identification with the organisation* (relationship and commitment to organisation) #4.5 *Other personal attributes* (learning styles, capacity, competency, motivation, etc.)
CFIR #5: Implementation process	#5.1 *Planning* processes for implementation #5.2 *Engagement* strategies (with opinion leaders, internal leaders, champions, external change agents, key stakeholders) #5.3 *Executing* according to implementation plan #5.4 *Reflecting and evaluating* (qualitative and quantitative feedback on progress)

### Study sample

Purposeful stratified sampling was used to seek a range of views of those involved in the implementation of PEACH™QLD and Go4Fun®. Sixteen study sites (eight per State), defined by State government geographic Health Service areas, were selected to reflect diversity regarding current level of program implementation (none, discontinued, repeated) and geographic location (as defined by the Accessibility/Remoteness Index of Australia: ARIA categories along a continuum of remoteness of major city, inner regional, outer regional, remote) [20] (Table 3). Sites were chosen in consultation with the respective State project managers using their current records of program delivery.

**Table 3:** Study sites by level of program implementation and geographic location

Level of program implementation	Geographic location				*Total sites*
	Major city	Inner regional	Outer regional	Remote	
PEACH™QLD sites (*n* = 8)					
No implementation	0	1	0	2	*3*
Discontinued implementation	1	0	0	0	*1*
Repeated implementation	1	1	2	0	*4*
Go4Fun® sites (*n* = 8)					
No implementation	0	0	0	0	*0*
Discontinued implementation	0	0	1	2	*3*
Repeated implementation	2	2	1	0	*5*
*Total sites*	*4*	*4*	*4*	*4*	*16*

In both States, State-level and site-level participants were interviewed. State-level participants included individuals in central agencies responsible for program development, funding, management, co-ordination and evaluation (*n* = 11). Site-level participants included individuals involved across the three program implementation roles of local program delivery (facilitating group sessions) (*n* = 12), co-ordination (recruitment of groups, scheduling of staff) (*n* = 15) and management (organisational support and resourcing) (*n* = 12). Forty-eight site-level participants were identified to reflect the three program implementation roles at each study site; however, not all participants could be contacted, and in some cases, a role had transitioned or was being performed by multiple people so additional participants were interviewed (Table 4). Data were not collected on the length of time participants had been in their role. No incentives were provided to participants.

**Table 4:** Study participants by State- and site-level program implementation roles

Study participants	Queensland (*n* = 27)			NSW (*n* = 23)		Total (*n* = 50)
State-level roles						
Funding	2			2		4
Co-ordination	1			1		2
Development	1			1		2
Evaluation	2			1		3
*State: total participants*	*6*			*5*		*11*
Site-level roles	None (3 sites)	Discontinued (1 site)	Repeated (4 sites)	Discontinued (3 sites)	Repeated (5 sites)	
Management	0	1	5	3	3	12
Co-ordination	3	1	4	3	4	15
Delivery	3	1	3	0	5	12
*Site: total participants*	*6*	*3*	*12*	*6*	*12*	*39*

### Data collection and analysis

The CFIR informed the development of an interview guide (Additional file 1). Questions and prompts were formulated to explore each of the constructs, excluding trialability. The interview was structured against key stages of program implementation including implementation decision-making, processes, support and evaluation. Interviews were conducted by telephone, audio recorded and transcribed verbatim. Signed consent was received prior to interview and verbal consent to record the interview was reconfirmed at the start of each interview. Interviews were conducted over a 2-month period, with an average duration of 30–45 min. The second author conducted all 50 interviews.

All transcripts were thematically coded against constructs using qualitative content analysis and the CFIR Coding Guide [21]. All transcripts were coded in NVIVO [22] by the second author, with a sub-sample double-coded by the first author (from Queensland) and external project officer (from New South Wales) to check for coding consistency. Coded constructs for each interview were scored using the CFIR Rating Rules, guided by the application of this approach by Damschroder and Lowery [18]. Numerical values (+2, +1, 0, − 1, − 2) were assigned to each coded construct, indicating the relative strength of a participant’s quote as a positive influence (‘enabler’) or negative influence (‘barrier’) on program implementation (Additional file 2). The second author scored all coded constructs, with a subset scored by the first author and project officer to compare for scoring consistency. Prior to scoring, the three coders met with CFIR developer, L. Damschroder, to check application of codes and scores.

CFIR construct scores for each interview were tabulated for site- and State-level interviews, with average scores being calculated as participant numbers varied across each category. Programmatic factors were identified by comparing and contrasting CFIR construct scores within each State to discern patterns between sites by level of program implementation (no implementation, discontinued, repeated) and their respective State-level central agency. Systemic factors were identified by comparing and contrasting CFIR construct between States to discern patterns between sites with repeated implementation and between State-level central agency perspectives. Distinguishing constructs were identified as those where sites with repeated implementation had a positive score and those with no or discontinued implementation had a negative score. Strength of the construct was based on the magnitude of the difference in scores. All results were peer reviewed by the Study Advisory Committee which comprised respective program funders, project managers and evaluators in both States, two implementation scientists from The Australian Prevention Partnership Centre which funded the study and the research team.

## Results

This research focused on identifying key factors that supported or inhibited repeated program implementation. Programs in both States were remarkably similar in their nature (Table 1). Each was evidence based, had good quality resources and training, was linked to their original inventors and consistent with guidelines for best practice [23]. The two programs differed in the extent to which implementation occurred. All health service study sites had been approached by their State provider to implement their respective program. In NSW, all sites (*n* = 8) had implemented Go4Fun®, of which three had subsequently discontinued implementation, with an understanding that should they be able to make groups viable in the future they could re-engage in the program. In Queensland, five sites implemented PEACH™QLD, of which one had subsequently discontinued implementation; and three sites had opted not to implement the program (Table 3). Indicative quotes to support quantitative data are presented in Additional file 2.

### Common site-level enablers and barriers

#### Influence of geographic location

Queensland covers a larger landmass than NSW, with a higher degree of remoteness (remote and very remote towns). In both States, all remote sites had either never implemented (Queensland) or had discontinued implementation (NSW), indicative of the challenges faced in relation to the degree of complexity (CFIR#1.6), costs (CFIR#1.8) and not meeting participant needs (CFIR#2.1). These barriers inhibited embedding the program into routine practice due to small population sizes and larger travelling distances which limited program recruitment, engagement and retention and created additional program resource distribution costs. Information on the costs of delivering the program in each State is not included in this paper. It is well established, however, that costs of providing and participating in services in remote areas of Australia are greater than those in urban areas even when the program itself is free to patients.

#### Influence of implementation model

Models of implementation varied between States with greater variability in Queensland than in NSW (Table 5), reflecting the different approaches of the two State governments. Program co-ordination through local Health Services occurred across all NSW sites, compared with only two Queensland sites. Program delivery using external, contracted staff or agencies was common across the majority of sites for both States. This model appeared to pose challenges for remote and outer regional areas, already experiencing an isolated, small and part-time workforce with a high turnover, who described implementation barriers relating to reduced identification of staff with the organisation (CFIR#4.4) and an absence of formally appointed internal implementation staff (CFIR#5.2.2). Barriers appeared inter-related, negatively affecting the quality of program session delivery and engagement and retention of participants*.*

**Table 5:** Model of implementation according to level of program implementation and geographic location by State

Model of implementation	Level of program implementation		
	Never (3 sites)	Discontinued (4 sites)	Ongoing (9 sites)
	QLD inner regional QLD remote QLD Remote		
a) Co-ordinated by health service; delivered by internal facilitators		QLD major city	QLD major city NSW inner regional
b) Co-ordinated by health service; delivered by contracted facilitators		NSW outer regional ^c,b^ NSW remote ^c,b^ NSW remote ^c,b^	NSW major city NSW major city NSW inner regional NSW outer regional
c) Co-ordinated and delivered by other agency			QLD outer regional
d) Delivered by contracted facilitators; no co-ordination role			QLD inner regional QLD outer regional ^c,d^

Footnotes indicate a transition from one delivery model to another

#### CFIR constructs

Across Queensland and NSW health service sites, irrespective of level of implementation, two constructs were common negative influences (‘barriers’)—complexity of implementation (CFIR#1.6) and meeting participant needs (CFIR#2.1). Complexity was primarily described as the lack of clarity regarding roles and responsibilities for childhood obesity management service delivery across the health service continuum, funding uncertainties, model of delivery and the program not aligning to the core business of the organisation. In both States, sites with repeated implementation identified the relative advantage (CFIR#1.3) of implementing the program over an alternative. Adaptability (CFIR#1.4) was a distinguishing characteristic between sites with repeated implementation and those that had discontinued, particularly in Queensland.

The distinguishing constructs between Queensland sites with repeated implementation and those in NSW were as follows: identification by program delivery staff with the organisation (CFIR#4.4), external policy and incentives (CFIR#2.4), supportive organisational culture (CFIR#3.3), implementation planning processes (CFIR#5.1) and external change agents (CFIR#5.2.4). These were all identified more strongly in NSW as supporting repeated implementation than in Queensland.

### Within-State comparisons

#### Queensland

In comparison to their local health service sites, Queensland central agencies rated one construct as a strong negative influence—complexity of implementation (CFIR#1.6)—and five constructs as moderate negative influences—acknowledgement of the need (tension) for change (CFIR#3.4.1), alignment with external policy and incentives (CFIR#2.4), program champions within the organisation (CFIR#5.2.3), evaluation and feedback processes (CFIR#5.4) and leadership engagement (CFIR#3.5.1). These sat across all CFIR domains, whereas the three constructs that were perceived as moderate positive influences all sat within the intervention characteristics domain—the intervention source (CFIR#1.1), evidence supporting the intervention (CFIR#1.2) and relative advantage that program delivery offered (CFIR#1.3) (Table 6).

**Table 6:**
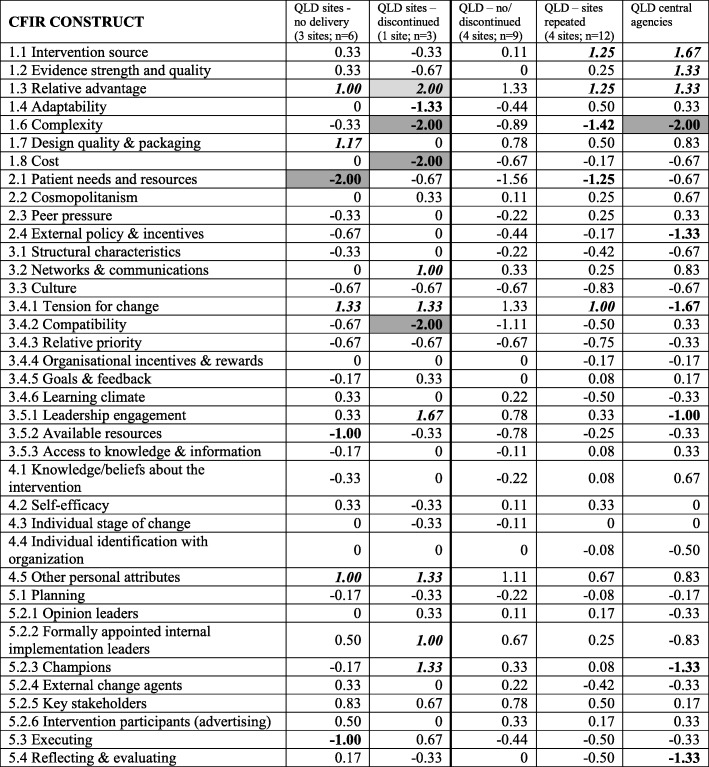
Ratings assigned to CFIR constructs for within-State comparisons between sites (according to level of implementation) and the central agencies in Queensland (QLD)

Perceived influence of constructs: bolded and shaded denotes strongly negative (-2.00); bolded denotes moderately negative (-1.00); italics, bolded and shaded denotes strongly positive (+2.00); italics and bolded denotes moderately positive (+1.00)

*1.5 Trailability was excluded as both programs have been trialed

When comparing CFIR construct scores between Queensland health service sites with discontinued and repeated implementation, strongly distinguishing constructs were source of the intervention (CFIR#1.1) and adaptability of implementation to meet local needs (CFIR#1.4). Moderately distinguishing constructs were evidence supporting the quality of the program (CFIR#1.2) and self-efficacy of the program facilitators (CFIR#4.2).

No strongly distinguishing constructs were identified when comparing CFIR construct scores between Queensland health service sites with repeated implementation and the perspectives of their central agencies. Moderately distinguishing constructs included acknowledgement of the need (tension) for change (CFIR#3.4.1), leadership engagement (CFIR#3.5.1) and program champions within the organisation (CFIR#5.2.3).

#### NSW

Across NSW health service sites, irrespective of level of implementation, two constructs were common negative influences—complexity of implementation (CFIR#1.6) and meeting participant needs (CFIR#2.1)—and three constructs were common positive influences—available networks and communication (CFIR#3.2), achievable implementation goals (CFIR#3.4.5) and formally appointed internal implementation leaders (CFIR#5.2.2). (Table 7).

**Table 7:**
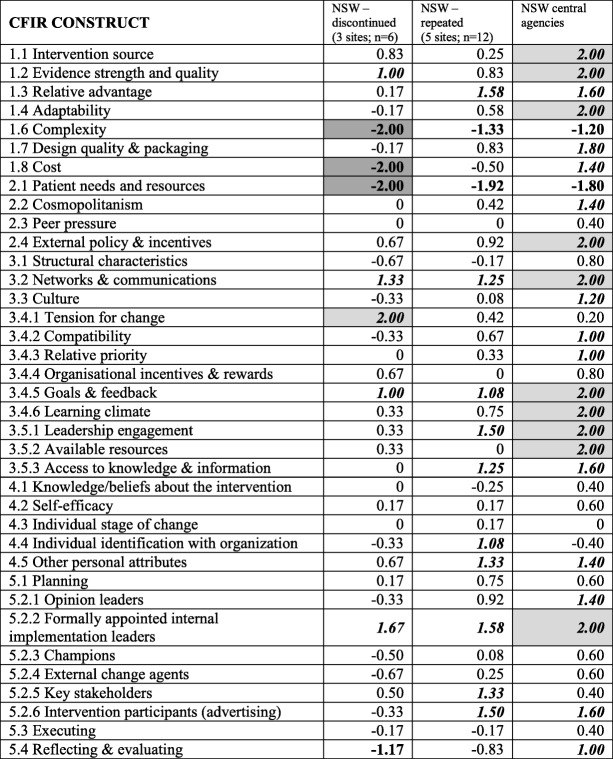
Ratings assigned to CFIR constructs for within-State comparisons between sites (according to level of implementation) and the central agencies in New South Wales (NSW)

Perceived influence of constructs: bolded and shaded denotes strongly negative (-2.00); bolded denotes moderately negative (-1.00); italics, bolded and shaded denotes strongly positive (+2.00); italics and bolded denotes moderately positive (+1.00)

*1.5 Trailability was excluded as both programs have been trialed

In comparison to their local health service sites, NSW State central agencies did not rate any CFIR constructs as strong negative influences; although consistent with the views of sites and Queensland, meeting participant needs (CFIR#2.1) and complexity of implementation (CFIR#1.6) were perceived as moderately negative. Ten constructs were rated as strong positive influences and a further 12 as moderate positive influences. These were particularly in the domains of intervention characteristics and inner setting.

When comparing CFIR construct scores between NSW health service sites with discontinued and repeated implementation, identification by program delivery staff with the organisation (CFIR#4.4) was a strongly distinguishing construct. Numerous moderately distinguishing constructs were apparent in relation to intervention adaptability (CFIR#1.4) and design and packaging (CFIR#1.7); organisational culture (CFIR#3.3) and compatibility with organisational goals (CFIR#3.4.2); engagement of opinion leaders (CFIR#5.2.1), program champions (CFIR#5.2.3) and external change agents (CFIR#5.2.4); and program promotion to intervention participants (CFIR#5.2.6).

No strongly distinguishing constructs were identified when comparing CFIR construct scores between NSW health service sites with repeated implementation and the perspectives of their central agencies. Moderately distinguishing constructs included intervention cost (CFIR#1.8) and intervention evaluation processes (CFIR#5.4).

### Between-State central agency comparisons

The comparison of health service sites with repeated implementation between States and central agency perspectives is described in Table 8 with illustrative quotes presented in Additional file 2.

**Table 8:**
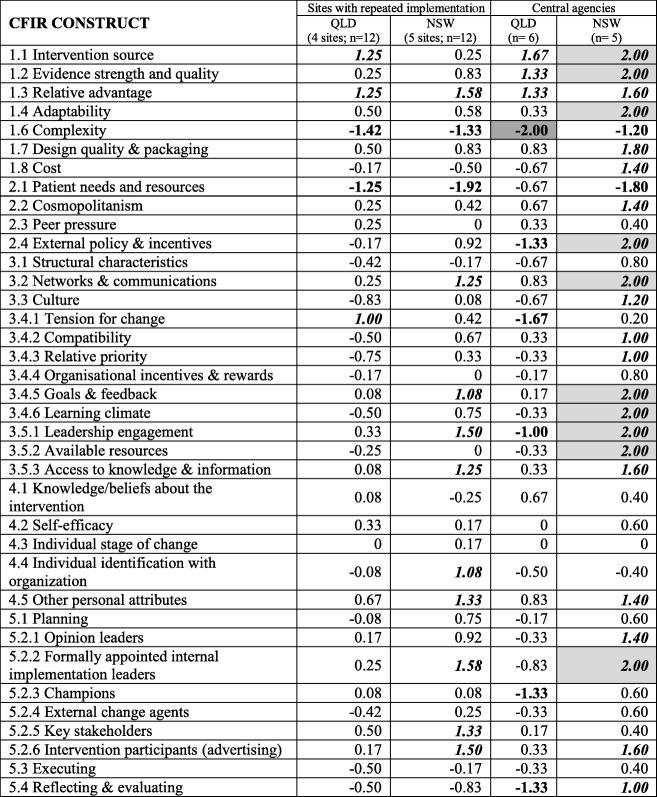
Ratings assigned to CFIR constructs for between-State comparisons for Queensland and New South Wales sites with repeated implementation and central agencies

Perceived influence of constructs: bolded and shaded denotes strongly negative (-2.00); bolded denotes moderately negative (-1.00); italics, bolded and shaded denotes strongly positive (+2.00); italics and bolded denotes moderately positive (+1.00)

*1.5 Trailability was excluded as both programs have been trialed

When comparing CFIR construct scores between QLD and NSW central agency perspectives, again, complexity of implementation (CFIR#1.6) was consistently identified as a negative influence. Three constructs, all within the intervention characteristics domain, were common positive influences—the intervention source (CFIR#1.1), evidence supporting the quality of the program (CFIR#1.2) and relative advantage that program delivery offered (CFIR#1.3). Compared to QLD, NSW central agencies had numerous distinguishing constructs, in particular, external policy and incentives (CFIR#2.4), supportive implementation learning climate (CFIR#3.4.6), leadership engagement (CFIR#3.5.1), available resources (CFIR#3.5.2) and formally appointed internal implementation leaders (CFIR#5.2.2). The presence of these implementation constructs were all seen as positive influences in NSW and their absence seen as a negative influence on implementation in Queensland.

## Discussion

This paper presents the perspectives of all those involved in the system of ongoing service provision to the families of overweight or obese children in the case study States. In doing so, it presents the complexity of service delivery as described by the CFIR constructs, how constructs present differently at system, and service levels, and the relationship between these. The decision to include 37 CFIR constructs followed a desire of both States’ teams to broadly explore the breadth of implementation characteristics rather than assume the importance of some over others. While numerous studies have explored the key determinants of successful program implementation using the CFIR [18, 24], few have examined this in the context of childhood weight management program implementation [15], and to our knowledge, none has explored the interplay between the layers of systems required for ‘at scale’ routine service delivery. This resulted, however, in a complex data set compounded by several constructs which overlapped rather than being discrete elements. Of interest was the extent to which there was consistency in the perceptions of different agencies and system actors, which was indicative of a joined-up, well-supported implementation system for a consistently available universal service for the management of childhood overweight and obesity. This discussion reflects on barriers and enablers that (i) were commonly identified by central agencies in both States and health service sites irrespective of level of implementation; (ii) distinguish sites with repeated, discontinued or no implementation; (iii) are inconsistently identified between central agencies and the sites in their State; and (iv) differ between Queensland and NSW overall.

### Barriers and enablers commonly identified by health service sites and central agencies in both States and irrespective of level of implementation

Irrespective of their level of implementation, both Queensland and NSW sites and central agencies strongly identified *complexity* of implementing an intervention to address childhood obesity as a barrier. Given that both States were offering a universal childhood weight management service for the first time, this was perhaps to be expected as it required significant re-orientation of organisations’ activities [25]. Reflective of the Australian health care system and its lack of clarity over the responsibility for the delivery of childhood weight management services, interviewees described complexity in terms of a lack of consensus regarding the stewardship for childhood weight management programs.

The issue of stewardship is an ongoing debate for countries, and while some governments are making child obesity a health priority, ‘obesity is not a distinct clinical problem, (therefore) there is no single clinical practice that ‘owns’ the condition’ [26], and management tends to be uncoordinated [27]. Philosophical differences regarding where childhood obesity management fits within the health care continuum, and alignment with the broader external policy environment, compromised repeated program delivery. This lack of clarity resulted in shifting ownership of the delivery of services, inconsistent availability of such services and extensive administrative resources needed to repeatedly engage service providers. In particular, there was a lack of appropriate clinical pathways and formal referral processes. National, State and private health service providers involved in the management of childhood overweight and obesity were not involved in these programs in a co-ordinated way. Systematic referral processes and timely follow-up are regarded as facilitators to participation, especially for vulnerable families [14, 28]. The persistence of this issue, despite repeated service delivery, suggests that for weight management services to become part of routine universal care, greater clarity regarding stewardship is still needed. For Australia, this requires system change beyond that controlled by States.

Meeting the needs of families and children was also seen as a common barrier to ongoing service delivery. This included needs resulting from the life stage, the health issue and its determinants, and geography. These were highlighted particularly with regard to families experiencing disadvantage and living in rural and remote areas. This is concerning given these families are already more likely to experience a higher burden of disease [2]. These challenges are consistently reported in the literature [29, 30], most notably the stigma of being overweight and denial of the issue amongst some parents [31] and the specific needs of families living in rural areas in relation to transportation difficulties, time constraints, limited access and availability of healthy foods, and lower socio economic position [32]. In both Queensland and NSW, their State-level response to families of children with overweight or obesity was limited to a one-size-fits-all program. A group-based community program is unlikely to meet the needs of all families, and it may require a range of service options being available.

### Barriers and enablers that distinguish sites with repeated, discontinued or no implementation

In both States, all health service sites in remote locations had either never implemented, discontinued or suspended program delivery. This is an important finding with respect to the transferability of implementation models and upscaling of programs to universal Statewide delivery if the original program has been developed and piloted in a different type of geographical area. The influence of contextual factors on program implementation within rural Australia is described in detail by Kozica et al. [33], who recommend that programs are developed as a suite of resources that allow for program flexibility and adaption to local needs and which can be easily and cost-effectively delivered with the assistance of volunteers and community champions.

### Barriers and enablers inconsistently identified between central agencies and the sites in their State

Key drivers of implementation from a programmatic perspective differed between States. *Program adaptability* to meet local needs and the importance of the *intervention source* were important to QLD sites and *identification by program delivery staff with the organisation* important to NSW sites. These site-level perspectives differed from those of their respective central agencies. QLD central agencies appeared not to prioritise the *need (tension) for change*, *leadership engagement* or *state-level program champions* compared with their respective sites with repeated implementation. NSW central agencies appeared to regard the *cost of intervention delivery* and *intervention evaluation processes* as less prohibitive compared with their respective sites with repeated implementation.

### Barriers and enablers that differ between Queensland and NSW overall

The evidence base and the quality of the support and resources of both programs were considered positive influences by all those involved in implementation and considered as providing a *relative advantage* to the organisation compared to the delivery of an alternate program. Both PEACH™ [8, 9] and Go4Fun®/MEND [11, 34, 35] have a long history underpinned by rigorous research. This supported practitioners and policy makers in advocating for implementation. There were challenges, however, in using an evidence-based program, including negotiating intellectual property and developing adaptations to extend reach and engagement. Adaptations were successful when pre-tested using an evaluation framework. Ongoing monitoring and review of program outcomes was important for the allocation of resources for ongoing program availability but needed to be considered alongside the burden of collecting data from families. The importance of childhood obesity as an urgent health issue was acknowledged at a State policy level within both Queensland [36] and NSW [37]; however, organisational acknowledgement of the need for a childhood obesity management program, assurances of the strength of the evidence base of the program and the provision of high-quality resources and support appeared to be insufficient to achieve sustained program delivery when implementation was associated with a high degree of complexity and costs, and limited leadership. This was evident in NSW where the *tension (need) for change* extended to State-level central agencies leading site-level program co-ordination with clearly articulated roles for the spectrum of implementation functions. This was strengthened further through dedicated funded positions for implementation staff, centralised service provision, defined program delivery targets, co-ordinated networks and communication for program implementation staff at site and State levels. Recent reports that childhood obesity prevalence remains high re-emphasise the urgency for countries to take definitive action to develop comprehensive national strategies with clearly articulated implementation frameworks, committed investment and allocated resources [38].

The importance of *leadership* was described as a strategy to address the complexity of childhood obesity intervention but seen as limited at a program and system level. This included the level of engagement needed from key stakeholders, opinion leaders, organisational champions and external change agents to generate and sustain program investment, awareness, recruitment and delivery. The role that leaders and transformational leadership can play in creating a supportive implementation climate conducive to program sustainment is well documented [18, 39–41]. Leadership at both a program and system level in the form of policy, procedure, reward systems, supportive championing to stakeholders, institutionalisation through secure funding and communication congruent across all levels have been associated with positive employee attitudes, motivation and performance in support of implementation.

### Strengths and limitations

This research attempted to identify organisational and system factors influencing successful implementation of childhood obesity management programs using a recognised definition and theoretical framework within the field of implementation science. Findings are therefore relevant to informing the integration of program implementation into routine practice.

Methodological limitations included locating adequate representation of interviewees for sites that had never commenced or had discontinued program delivery, with some positions unavailable for interview. All remote sites had never implemented or had discontinued implementation; therefore, findings in relation to rural sites should be extrapolated with caution.

The CFIR provided a comprehensive typology to explore potential barriers and enablers to implementation in a consistent manner. Although coding and rating was undertaken by two independent researchers to minimise subjective bias, an iterative review process would have been required to eliminate this bias. This resource and time-intensive task was not possible given the large sample size. Coding may have also been influenced by the large sample size and number of CFIR constructs, with several constructs overlapping.

### Impact of research on field of implementation science

This research was unique in being able to compare two Australian State governments’ attempts at the provision of a universal childhood obesity management service. This is an important progression of the evidence base in the field of childhood obesity management, revealing the complexity of embedding a program as universal routine service delivery through a public health service system.

This paper used the CFIR as at the time there were no consolidated sustainability frameworks available, and the CFIR was considered most appropriate to examine factors influencing implementation across a continuum of none to ongoing. Given the recent emergence of a consolidated sustainability framework [42], it may be useful to apply these proposed constructs to the findings from this study and contrast and compare these two frameworks.

## Conclusion

The WHO describes a well-functioning health system as one that delivers equitable access to effective, safe and quality interventions ‘to those that need them, when and where needed’, with sufficient staffing, adequate funding, timely information on performance, and leadership and governance supported by strategic policy [43]. Similar criteria are recommended for the implementation of community-based interventions to address obesity [44, 45]. The National Institute for Health and Care Excellence (NICE) [46] provides comprehensive guidance for the provision of lifestyle weight management services to children and young people, with a similar emphasis on raising awareness of the program amongst providers, health and other professionals and community organisations; establishing formal referral processes; providing ongoing support and training to staff; and monitoring and evaluation. Similarly, a recent systematic review exploring the sustainability of initiatives delivered within the health care setting identified 40 individual constructs, of which six were consistently applied, irrespective of the type of initiative or the setting, namely—general resources, demonstrating effectiveness, monitoring progress, stakeholder participation, integration into existing programs and policies, and training and capacity building [42].

There is remarkable alignment between these criteria and the enablers and barriers identified in relation to implementation of childhood obesity management programs in Queensland and NSW, Australia. Understanding these enablers and barriers to implementation ‘at scale’ is imperative to inform future planning and investment by Australia to meet its commitment, as a member State to the World Health Assembly, of providing sustainable childhood obesity management services through routine universal health care [5].

## Additional files

Additional file 1: Interview questions based on CFIR constructs. (PDF 315 kb)

Additional file 2: CFIR construct ratings with indicative quotes (see comment blocks within colour-coded blocks). (XLSX 25 kb)

## Abbreviations

CFIR: Consolidated Framework for Implementation Research
NPAPH: National Partnership Agreement on Preventative Health
NSW: New South Wales
PEACH™: Parenting, Eating, Activity for Child Health
PEACH™QLD: The implementation of PEACH™ in Queensland from 2013 to 2016
QLD: Queensland
QUT: Queensland University of Technology
WHO: World Health Organization
