# Supplementary material for: A description of health care system factors in the implementation of universal weight management services for children with overweight or obesity: case studies from Queensland and New South Wales, Australia
Source: Implement Sci. 2018 Aug 7;13:109. doi: 10.1186/s13012-018-0801-2 (PMC6081901; doi:10.1186/s13012-018-0801-2)
Supplement: Supplementary file 1 — Interview questions based on CFIR constructs. (PDF 315 kb) [file 13012_2018_801_MOESM1_ESM.pdf]

PROPOSED INTERVIEW QUESTIONS BASED ON CFIR CONSTRUCTS

Please describe your usual role within the organization.
Please describe your role in relation to the intervention.

DECISION TO IMPLEMENT

How was the decision made:
1. How did your setting/organization become involved in implementing the intervention? (Im)
Who decided that the intervention would be implemented in your setting/organization? (IS)
Were you involved in the decision to implement the intervention? In what ways? (IS)
How did/do you feel about the intervention being implemented in your setting/organization? (KB)
What did/do staff/leaders in your setting think of the intervention? (ES)

Why was the decision made:
2. Why do you think the decision was made to implement the intervention in your setting? (IS)
To what extent did your setting/organization see a need to implement the intervention? (TC)
Was this to meet an organizational goal or the needs of people served by the organization? (TC)
Were there any kinds of performance measures, policies, regulations or guidelines that influenced the decision to implement the intervention in your setting? (EPI)
Were there any financial or other incentives that influenced the decision to implement the intervention in your setting? (EPI)

Choice of intervention:
3. What kind of information/evidence are you aware of that shows that this intervention would work in your setting? (ES)
How important was/is it to you that the intervention has good quality evidence to back it up? (ES)
Are you aware of any other settings/organizations that have implemented this intervention? Describe (PP)
Did this influence how your setting/organization implemented the intervention? In what ways? (PP)
Do you see it as an advantage that your setting/ organization implemented this intervention? (PP)
Have you been involved in implementing similar interventions in your setting? Describe (RA)
How did it compare to this intervention (advantages and disadvantages)? (RA)
Is there another intervention that you think staff in your setting would prefer to implement? What is this and why would they prefer this alternative? (RA)

Organizational/management support:
4. Do you think the culture of your work unit/setting/organization affected implementation of the intervention? In what ways? Cu)
How would you describe the general level of willingness (receptivity) within your setting to implementing the intervention? (IC)
What level of support/endorsement was provided by senior management / leaders for the implementation of the intervention? (LE)
How was the intervention communicated to leaders/senior managers within your setting/ organization? (OL)
To what extent do you think leaders/senior managers influenced others within the organization to implement the intervention? (OL)

Resourcing:
5. Were adequate resources provided to implement the intervention? Eg: staffing, preparation time, space, equipment, etc (R)
Were there any incentives offered within your setting/ organization to help ensure the intervention was implemented successfully? (OIR)
Did these have an impact? In what ways? (OIR)
Were there any costs to your setting in implementing the intervention? If yes, were any of these costs unexpected? ($)

IMPLEMENTATION SUPPORT

Implementer characteristics:
6. Who led/leads the implementation of the intervention in your setting/organization? (Appointed/Volunteered?) (Im)
Did the implementer have the sufficient authority to do what was necessary to implement the intervention? (Im)
Did the implementer have adequate knowledge about the intervention to implement it successfully? (KB)
Did the implementer have the required attributes to implement the intervention? (Im)
What personal attributes do you think were useful to implement the intervention? Eg: motivation, background knowledge, capacity (PA)
How confident did the implementer and associated work colleagues feel about implementing the intervention? Why? (SB)

Implementer support:
7. What kinds of information/training were provided to the implementer/ colleagues to support the implementation of the intervention? Helpful? (KI)
Did the implementer know who to ask for assistance about the intervention? Was assistance timely and helpful? (KI)
How would you describe the quality and appropriateness of any supports (online resources, marketing materials, toolkit) that were available to implement the intervention? (DQ) Were these supports readily accessible to staff? (DQ) Were they helpful? (DQ)
What level of support did the implementer receive from leaders / senior management to implement the intervention in your setting? (IdO)

Internal support:
8. Apart from the 'implementer' were there other people within your setting/organization who were champions of the intervention? (Ch)
What was their contribution to the implementation of the intervention? (Ch)

External support:
9. Did you have other people outside of your setting/ organization that helped with implementing the intervention? (CA)
What was their contribution to the implementation of the intervention? (CA)

Communication:
10. How do/did you and your colleagues typically communicate within your setting about the intervention? Formal, informal? Frequency? (NC)
What kind of information sharing do you have with professionals outside your setting/organization about the intervention? (Co)
Were/are you encouraged to network and share information about the intervention outside of your setting/ organization? (Co)

Competing priorities:
11. When implementing the intervention, were there any other high priority activities taking place at the same time? Describe (RP)
Did this affect implementation of the intervention? In what ways? (RP)
Did this affect implementation of other high priority activities? In what ways? (RP)
How important do you think it was to implement the intervention compared to these other priorities? (RP)

Planning:
12. How would you describe the planning process used to implement the intervention in your setting/ organization? (PL)
What was your role in the planning process? (PL)
Was the intervention implemented as per the proposed planned (if there was one)? In what ways? (Exe)

OUTCOMES

Implementation:
13. How complicated was it to implement the intervention in your setting/organization? (C)
What kinds of changes were needed so that the intervention could be implemented in your setting? Infrastructure (changes in scope of practice / formal policies / information systems / other (SC); other (Ad)
Who decided whether changes were needed and what was the process for making these changes? (Ad)
Was there support to make these changes? (Ad)
To what extent do you feel you can suggest new ideas and try new things to make improvements in your setting/organization? (LC)
In retrospect, were there changes you wish had/had not been made? (Ad)
Was the intervention seen as part of routine practice or a departure from previous practices? In what way? (C) / How well did the intervention fit with existing with practices and processes? Did issues/complications arise? (Com)
Has the intervention been integrated into current practice? In what ways? (Com)
Did you integrate the implementation of the intervention into your usual practice? How? (SoC)

Meeting intervention goals:
14. Did your setting/organization set any goals related to the implementation of the intervention? (GF)
How well does/did these goals align with the values and norms (goals) of your setting/organization? (GF)/(Com)
How were these goals monitored for progress? (GF) / How did you assess if implementation was being effective? Goals were being met? (Eva)
How was progress communicated? (GF)
Were any feedback reports about the implementation of the intervention compiled/disseminated? Describe (Eva)
Do you have any comments about the evaluation information that was collected as part of the intervention? (Eva)
Did you collect any additional information locally? For what purpose? (Eva)

Meeting target audience needs:
15. To what extent do you think the intervention met the needs and preferences of the target audience? In what ways? (PN)
Was the intervention changed in any way to meet these needs and preferences? In what ways? (PN)
What barriers did the target audience face to participate in the intervention? Were these addressed and if so, how? (PN)
How was the intervention communicated/promoted to the target audience? (IP)
How was the intervention communicated/promoted to key stakeholders outside your setting/organization? (KS)
To what extent do you think key stakeholders influenced the target audience to enroll in the intervention? (referrals) (KS)
Do you think the intervention has been effective in your setting? Why do you say that? (KB)

Access to Knowledge & Information (KI)
Adaptability (Ad)
Available Resources (R)
Champions (Ch)
Compatibility (Com)
Complexity (C)
Cosmopolitanism (Co)
Cost ($)
Culture (Cu)
Design Quality & Packaging (DQ)
Engaging
Evidence Strength & Quality (ES)
Executing (Exe)
External Change Agents (CA)
External Policy & Incentives (EPI)
Formally Appointed Internal Implementation Leaders (Im)
Goals and Feedback (GF)
Implementation Climate (IC)
Individual Identification with Organization (IDO)
Individual Stage of Change (SoC)
Intervention Participants (IP)
Intervention Source (IS)
Key Stakeholders (KS)
Knowledge & Beliefs about the Intervention (KB)
Leadership Engagement (LE)
Learning Climate (LC)
Networks & Communications (NC)
Opinion Leaders (OL)
Organizational Incentives & Rewards (OIR)
Other Personal Attributes (PA)
Patient Needs & Resources (PN)
Peer Pressure (PP)
Planning (PL)
Reflecting & Evaluating (Eva)
Relative Advantage (RA)
Relative Priority (RP)
Self-efficacy (SB)
Structural Characteristics (SC)
Tension for Change (TC)

Consolidated Framework for Implementation Research (CFIR) Constructs

Construct	Short Description	CFIR example questions	Interview guides
**1. INTERVENTION CHARACTERISTICS**			
1.1 Intervention Source (IS)	Perception of key stakeholders about whether the intervention is externally or internally developed.	1.1.1 Who developed the intervention?<br>1.1.2 What is your opinion of this group/ individual?<br>1.1.3 Why is this intervention being implemented in your organization/setting?<br>1.1.4 Who decided to implement the intervention?<br>1.1.5 How was the decision made to implement the intervention?	Who decided that the intervention would be implemented in your setting? (IS)<br>What was your involvement in the decision to implement the intervention in your setting? (IS)<br>Why do you think the decision was made to implement the intervention in your setting? (IS)
1.2 Evidence Strength & Quality (ES)	Stakeholders' perceptions of the quality and validity of evidence supporting the belief that the intervention will have desired outcomes.	1.2.1 What kind of information/evidence are you aware of that shows whether or not the intervention works/worked in your organization/setting? (Practice guidelines? Published literature?)<br>1.2.2 How does this knowledge affect your perception of the intervention?<br>1.2.3 What do influential staff / leaders in your organization/setting think of the intervention?<br>1.2.4 What do other staff (admin) think of the intervention?<br>1.2.5 What kind of supporting evidence/proof is needed about the effectiveness of the intervention to get staff on board? (Co-workers? Leaders? Admin?)	What kind of information/evidence are you aware of that shows that this intervention would work in your setting? (ES)<br>How important is it to you that the intervention has good quality evidence to back it up? (ES)<br>What do staff/leaders in your setting think of the intervention? (ES)
1.3 Relative Advantage (RA)	Stakeholders' perception of the advantage of implementing the intervention versus an alternative solution.	1.3.1 How does the intervention compare to other similar existing programs in your organization/setting?<br>1.3.2 What advantages/disadvantages does the intervention have compared to these other programs?<br>1.3.3 Is there another intervention that people would rather implement? Can you describe that intervention? Why would people prefer this alternative?	Have you been involved in implementing similar interventions in your setting? What was this and how did it compare to this intervention (advantages and disadvantages)? (RA)<br>Is there another intervention that you think staff in your setting would prefer to implement? What is this and why would they prefer this alternative? (RA)
1.4 Adaptability (Ad)	The degree to which an intervention can be adapted,	1.4.1 What kinds of changes/alterations did you need to make to the intervention so it works/	What kinds of changes did you make to the intervention so it worked (effectively) in your setting? (Ad)

	tailored, refined, or reinvented to meet local needs.	worked effectively in your organization/ setting?<br>1.4.2 Who decided whether changes were needed?<br>1.4.3 What was the process for deciding that changes were needed?<br>1.4.4 How did you know it was appropriate to make these changes?<br>1.4.5 Are there components of the intervention that should not be changed/ altered? Which ones are these?	Who decided whether changes were needed and what was the process for making these changes? (Ad)<br>Was there support to make these changes? (Ad)<br>In retrospect, were there changes you wish had/had not been made? (Ad)
1.5 Trialability	The ability to test the intervention on a small scale in the organization, and to be able to reverse course (undo implementation) if warranted.	1.5.1 Was the intervention piloted prior to full-scale implementation? Why/why not?<br>1.5.2 Was the pilot helpful?	n/a (pilot/RCT undertaken prior to state roll out)
1.6 Complexity (C)	Perceived difficulty of implementation, reflected by duration, scope, radicalness, disruptiveness, centrality, and intricacy and number of steps required to implement.	1.6.1 How complicated is the intervention? (duration, scope, intricacy, steps involved)<br>1.6.2 Does the intervention reflect a clear departure from previous practices?	How complicated was it to implement the intervention? (C)<br>Was the intervention seen as part of routine practice or a departure from previous practices? In what way? (C)
1.7 Design Quality & Packaging (DQ)	Perceived excellence in how the intervention is bundled, presented, and assembled.	1.7.1 What supports (online resources, marketing materials, toolkit) are available to help you implement the intervention? How do you access these materials?<br>1.7.2 How do these supporting materials affect implementation of the intervention in your organization/setting?<br>1.7.3 What is your perception of the quality of the supporting materials? Why?	How would you describe the quality and appropriateness of any supports (online resources, marketing materials, toolkit) that were available to help you implement the intervention? (DQ)<br>Were these supports readily accessible to you?<br>Were they helpful? (DQ)
1.8 Cost ($)	Costs of the intervention and costs associated with implementing the intervention including investment, supply, and opportunity costs.	1.8.1 What costs are incurred to implement the intervention?<br>1.8.2 What costs were considered when deciding to implement the intervention?	Were there any costs to your setting in implementing the intervention? If yes, were any of these costs unexpected? ($)

Construct	Short Description	CFIR example questions	Interview guides
**2. OUTER SETTING**			
2.1 Patient Needs & Resources (PN)	The extent to which patient needs, as well as barriers and facilitators to meet those needs, are accurately known and prioritized by the organization.	2.1.1 To what extent are staff / leadership aware of the needs and preferences of the people who participate/d in the intervention?<br>2.1.2 To what extent were the needs and preferences of these people considered when deciding to implement the intervention? Can you describe specific examples? Was the intervention altered to meet these needs and preferences?<br>2.1.3 How well do you think the intervention meets the needs of the people who participate in the intervention? In what ways? (eg: help with self management; improved access; reduced time)<br>2.1.4 How well do/did participants respond to the intervention?<br>2.1.5 What barriers do/did people face to participate in the intervention?<br>2.1.6 Do you elicit information from participants regarding their experiences with the intervention? What specific information / stories have you heard?	To what extent do you think the intervention met the needs and preferences of the target audience? In what ways? (PN)<br>Was the intervention changed in any way to meet these needs and preferences? In what ways? (PN)<br>What barriers did the target audience face to participate in the intervention? Were these addressed and if so, how? (PN)
2.2 Cosmopolitanism (Co)	The degree to which an organization is networked with other external organizations.	2.2.1 To what extent do you network with colleagues/others in similar professions outside your organization/setting?<br>2.2.2 What kind of information exchange do you have with others outside your organization/setting generally about your profession/related to the intervention? (professional networks, conferences, etc)<br>2.2.3 To what extent does your organization/ setting encourage you to network with colleagues outside your organization/setting?	What kind of information sharing do you have with professionals outside your setting/organization related to the intervention? (Co)<br>Are you encouraged to network and share information about the intervention outside of your setting/ organization? (Co)
2.3 Peer Pressure (PP)	Mimetic or competitive pressure to implement an intervention; typically because most or other key peer or competing organizations have already implemented or are in a bid for a competitive edge.	2.3.1 Do you know about other organizations/ settings/work unit that have implemented the intervention / similar program?<br>2.3.2 To what extent are they implementing the intervention?	Are you aware of any other setting/organization that have implemented this intervention? Describe (PP)<br>Did this influence how your setting/organization implemented the intervention? In what ways? (PP)<br>Do you see it as an advantage that your setting/ organization has implemented this intervention? (PP)

		2.3.3 How has this influenced the decision to implement the intervention within your organization/setting/work unit?<br>2.3.4 How has this affected the support for implementing the intervention within your organization/setting/work unit?<br>2.3.5 To what extent would implementing the intervention provide an advantage for your organization/setting/work unit compared to other organizations/settings/work units?	
2.4 External Policy & Incentives (EPI)	A broad construct that includes external strategies to spread interventions, including policy and regulations (governmental or other central entity), external mandates, recommendations and guidelines, pay-for-performance, collaboratives and public or benchmark reporting.	2.4.1 What kinds of performance measures, policies, regulations or guidelines influence the decision to implement the intervention?<br>2.4.2 How does/did implementation of the intervention assist your organization/setting's ability to meet these measures?<br>2.4.3 What kind of financial or other incentives influenced the decision to implement the intervention?<br>2.4.4 How does/did implementation of the intervention assist your organization/setting's ability to receive these incentives?	Were there any kinds of performance measures, policies, regulations or guidelines that influenced the decision to implement the intervention in your setting? (EPI)<br>Were there any financial or other incentives that influenced the decision to implement the intervention in your setting? (EPI)

Construct	Short Description	CFIR example questions	Interview guides
**3. INNER SETTING**			
3.1 Structural Characteristics (SC)	The social architecture, age, maturity, and size of an organization.	3.1.1 How does the infrastructure of your organization/setting facilitate the implementation of the intervention?<br>3.1.2 How does the infrastructure of your organization/setting hinder the implementation of the intervention?<br>3.1.3 How have you worked around structural challenges?<br>3.1.4 What kinds of infrastructure changes were needed to accommodate the intervention? (changes in scope of practice / formal policies / information systems / other)<br>3.1.5 What kinds of approvals were needed? Who made these approvals? What process was needed to make these changes?	What kinds of infrastructure changes were needed so that the intervention could be implemented in your setting? Eg: changes in scope of practice / formal policies / information systems / other<br>How were these changes made? (SC)
3.2 Networks & Communications (NC)	The nature and quality of webs of social networks and the nature and quality of formal and informal communications within an organization.	3.2.1 How would you describe your working relationships with your colleagues? In your work unit? In other units?<br>3.2.2 To what extent do you get together with colleagues outside of work? To talk about work? Just for fun?<br>3.2.3 Do you meet with a team of people? Who is part of this team? How often do you meet? Formally or informally?<br>3.2.4 How would you describe your working relations with leaders? Supervisors (yours and of others)?<br>3.2.5 How would you describe your working relationship with influential stakeholders?<br>3.2.6 Are meetings, such as staff meetings, held regularly? Who typically attends? Do you typically attend? How often are they held? Agenda? Helpful?<br>3.2.7 How do you typically find out about new information/initiatives/issues/staff achievements/etc?<br>3.2.8 When you need to get something done, who are your go-to people?	How do you and your colleagues typically communicate within your setting about the intervention? Formal, informal? Frequency? (NC)

3.3 Culture (Cu)	Norms, values, and basic assumptions of a given organization.	3.3.1 How would you describe the culture of your organization/setting/work unit? Do you feel your work unit culture is different from the overall organization? In what ways?<br>3.3.2 How do you think the culture of your organization/setting/work unit does/did affect implementation of the intervention?<br>3.3.3 To what extent are new ideas embraced and used to make improvements in your organization/setting/work unit?<br>3.3.4 To what extent would you characterize the culture of your organization/setting/work unit as<br>a) a friendly workplace where leaders act like mentors, facilitators and team-builders (doing things together)<br>b) a structured and formalized workplace where leaders act like coordinators, monitors and organizers (doing things right)<br>c) a dynamic workplace with leaders that stimulate intervention (doing things first)<br>d) a competitive workplace with leaders like hard drivers, producers and competitors (doing things fast)	How would you describe the culture of your work unit/setting/organization? (Cu)<br>How do you think the culture of your work unit/setting/organization affected implementation of the intervention? (Cu)
3.4 Implementation Climate (IC)	The absorptive capacity for change, shared receptivity of involved individuals to an intervention, and the extent to which use of that intervention will be rewarded, supported, and expected within their organization.	3.4.1 What is the general level of receptivity in your organization/setting to implementing the intervention? Why?	How would you describe the general level of willingness (receptivity) within your setting to implementing the intervention? (IC)
3.4.1 Tension for Change (TC)	The degree to which stakeholders perceive the current situation as intolerable or needing change.	3.4.1.1 Is there a strong need for the intervention? Why/ not? Do others see a need for the intervention?<br>3.4.1.2 How essential is this intervention to meet the needs of intervention participants or other organizational goals?<br>3.4.1.3 Will this intervention fill current gaps?	To what extent did your setting/organization see a need to implement the intervention? (TC)<br>Was this to meet an organizational goal or the needs of people served by the organization? (TC)
3.4.2 Compatibility (Com)	The degree of tangible fit between meaning and values attached to the intervention by involved individuals, how those align with	3.4.2.1 How well does the intervention fit with your values and norms and those within your organization?	How well did the intervention align to the values and norms (goals) of your setting/organization? (Com)

	individuals' own norms, values, and perceived risks and needs, and how the intervention fits with existing workflows and systems.	3.4.2.2 How well does the intervention fit with existing work processes and practices in your setting? Have issues/complications arisen?<br>3.4.2.3 Describe how well the intervention has been integrated into current processes? Any conflicts with current programs/processes?<br>3.4.2.4 Has the intervention replaced / complimented a current program/process? In what ways?	How well did the intervention fit with existing with practices and processes? Did issues/complications arisen? (Com)<br>Has the intervention been integrated into current processes? In what ways? (Com)<br>Has the intervention replaced / complimented a current program/process? In what ways? (Com)
3.4.3 Relative Priority (RP)	Individuals' shared perception of the importance of the implementation within the organization.	3.4.3.1 What kinds of high priority initiatives are already happening in your setting?<br>3.4.3.2 What is the priority of implementing the intervention relative to these? Conflicts?<br>3.4.3.3 Will implementing the intervention help/hinder achieving these priorities?<br>3.4.3.4 Will implementation be delayed due to other priorities?<br>3.4.3.5 How important do you think it is to implement the intervention compared to other priorities?<br>3.4.3.6 How do you juggle competing work priorities and implementing the intervention? How do your colleagues do this?	When implementing the intervention, were there any other high priority activities taking place at the same time? Describe (RP)<br>How was implementation of the intervention affected? How was implementation of other high priority activities affected? (RP)<br>How important did you think it was to implement the intervention compared to the other priorities? (RP)
3.4.4 Organizational Incentives & Rewards (OIR)	Extrinsic incentives such as goal-sharing awards, performance reviews, promotions, and raises in salary, and less tangible incentives such as increased stature or respect.	3.4.4.1 What kinds of incentives are there to help ensure implementation of the intervention is successful?<br>3.4.4.2 What is your motivation for wanting to help ensure implementation s successful?<br>3.4.4.3 Are you aware of any recognition that is related to implementing the intervention? Can you describe them? Will they be targeted to individuals/teams/work units?	Were there any incentives offered within your setting/ organization to help ensure the intervention was implemented successfully? (OIR)<br>Did these have an impact? In what ways? (OIR)
3.4.5 Goals and Feedback (GF)	The degree to which goals are clearly communicated, acted upon, and fed back to staff, and alignment of that feedback with goals.	3.4.5.1 Does/did you/your work unit/ setting/organization set goals related to the implementation of the intervention? What?<br>3.4.5.2 To what extent does your organization/ setting set goals for current programs? How are these communicated? Can you give an example?<br>3.4.5.3 To what extent are goals monitored for progress? What type of information? Who is informed and how?	Did your setting/organization set any goals related to the implementation of the intervention? (GF)<br>How well did these goals align with the values and norms (goals) of your setting/organization? (GF)<br>How were these goals monitored for progress? How was progress communicated? (GF)

		3.4.5.4 Do you get any feedback reports about your work? In what form? Helpful? How often? From whom?<br>3.4.5.5 How does implementation of the intervention align with other organizational goals?	
3.4.6 Learning Climate (LC)	A climate in which: a) leaders express their own fallibility and need for team members' assistance and input; b) team members feel that they are essential, valued, and knowledgeable partners in the change process; c) individuals feel psychologically safe to try new methods; and d) there is sufficient time and space for reflective thinking and evaluation.	3.4.6.1 Can you describe a recent quality improvement initiative or an implementation of a new program? Motivation to implement it? Major milestones along the way? What factors helped make it a success? Who were the key players? What was your involvement? Were people happy with the outcome? How were leaders involved – who. Roles, helpful?<br>3.4.6.2 If you saw a problem in your setting, what would you do to resolve it?<br>3.4.6.3 To what extent do you feel like you can try new things to improve your work processes? Do you feel you have the time and energy to think about ways to improve things? Feel valued/respected by your supervisor for the role you play? What role does your supervisor play?	To what extent do you feel you can suggest new ideas and try new things to make improvements in your setting/organization? (LC)
3.5 Readiness for Implementation	Tangible and immediate indicators of organizational commitment to its decision to implement an intervention.	** see below	
3.5.1 Leadership Engagement (LE)	Commitment, involvement, and accountability of leaders and managers with the implementation.	3.5.1.1 What level of support/endorsement have you seen or heard from leaders? Who are they? How has this affected implementation?<br>3.5.1.2 What level of involvement has leadership within your organization had so far with the implementation? Who are they?<br>3.5.1.3 What level of support have they provided you? How?<br>3.5.1.4 What kind of support or actions can you expect from leaders within your organization to help make implementation successful? Who are they?<br>3.5.1.5 What types of barriers might they create?	What level of support/endorsement did you see/do you think was provided by senior management / leaders for the implementation of the intervention in your setting? Can you describe any examples? (LE)

3.5.2 Available Resources (R)	The level of resources dedicated for implementation and on-going operations, including money, training, education, physical space, and time.	3.5.2.1 Do you expect to have sufficient resources to implement and administer the intervention? What resources are you counting on? Would like to receive?<br>3.5.2.2 Will they be easy to procure? Who will assist with procurement? Challenges encountered?	Were adequate resources provided to implement the intervention? Eg: staffing, preparation time, space, equipment, etc (R)
3.5.3 Access to Knowledge & Information (KI)	Ease of access to digestible information and knowledge about the intervention and how to incorporate it into work tasks.	3.5.3.1 What kind of training was/is provided to you/your colleagues to implement the intervention? Helpful?<br>3.5.3.2 What kind of information/materials about the intervention was/is provided to you? Others in your organization? Helpful? Timely? Sufficient? Relevant?<br>3.5.3.3 Who do you ask if you have questions about the intervention? How available are they?	What kinds of information/training were provided to you/your colleagues to support the implementation of the intervention? Helpful? (KI)<br>Did you know who to ask if you to had questions about the intervention? Were questions answered effectively and in a timely manner? (KI)

Construct	Short Description	CFIR example questions	Interview guides
**4. CHARACTERISTICS OF INDIVIDUALS**			
4.1 Knowledge & Beliefs about the Intervention (KB)	Individuals' attitudes toward and value placed on the intervention as well as familiarity with facts, truths, and principles related to the intervention.	4.1.1 What do you know about the intervention or its implementation?<br>4.1.2 Do you think the intervention has been effective in your organization/setting? Why/not?<br>4.1.3 How do you feel about the intervention being implemented in your organization/setting? (anticipation, stress, enthusiasm) Why?<br>4.1.4 At what stage of implementation is the intervention in your organization/setting? How do you think it is going? Why do you say that?	Do you feel you had adequate knowledge about the intervention to implement it successfully? (KB)<br>Do you think the intervention has been effective in your setting? Why do you say that? (KB)<br>How did you feel about the intervention being implemented in your setting/organization? (KB)
4.2 Self-efficacy (SB)	Individual belief in their own capabilities to execute courses of action to achieve implementation goals.	4.2.1 How confident are you that you will be able to successfully implement the intervention? Why?<br>4.2.2 How confident do you think your colleagues feel about implementing the intervention? Why?	How confident did you feel about implementing the intervention? Why? (SB)<br>How confident do you think your work colleagues felt about implementing the intervention? Why? (SB)
4.3 Individual Stage of Change (SoC)	Characterization of the phase an individual is in, as he or she progresses toward skilled, enthusiastic, and sustained use of the intervention.	4.3.1 How prepared are you to implement the intervention?<br>a) knowledge of key aspects<br>b) has a positive view of and discusses the intervention with others<br>c) intends to seek additional information<br>d) seeks additional information<br>e) recognizes benefits, integrated into routine practice, promotes to others	Did you integrate the implementation of the intervention into your usual practice? How? (SoC)
4.4 Individual Identification with Organization (IDO)	A broad construct related to how individuals perceive the organization, and their relationship and degree of commitment with that organization.	** responses to other questions may be (double) coded here	What level of support did you receive from leaders / senior management to help you implement the intervention? (IDO)
4.5 Other Personal Attributes (PA)	A broad construct to include other personal traits such as tolerance of ambiguity, intellectual ability, motivation, values, competence, capacity, and learning style.	** responses to other questions may be (double) coded here	What personal attributes do you think were useful to you to implement the intervention? Eg: motivation, background knowledge, capacity (PA)

Construct	Short Description	CFIR example questions	Interview guides
**5. PROCESS**			
5.1 Planning (PL)	The degree to which a scheme or method of behavior and tasks for implementing an intervention are developed in advance, and the quality of those schemes or methods.	5.1.1 Who is involved in the planning process to implement the intervention? Appropriate people? Engaged?<br>5.1.2 What is your role in the planning process?<br>5.1.3 Do you have a plan in place to implement the intervention?<br>5.1.4 Can you describe this plan? How detailed? Who knows about it? Realistic?<br>5.1.5 What role has your plan played during implementation of the intervention? Progress tracking? Logging revisions, barriers, errors?	How would you describe the planning process used to implement the intervention in your setting/ organization? (PL)<br>What was your role in the planning process? (PL)
5.2 Engaging	Attracting and involving appropriate individuals in the implementation and use of the intervention through a combined strategy of social marketing, education, role modeling, training, and other similar activities.	** see below	
5.2.1 Opinion Leaders (OL)	Individuals in an organization who have formal or informal influence on the attitudes and beliefs of their colleagues with respect to implementing the intervention.	5.2.1.1 Who do you think are the key influencers to get onboard with the **implementation** of this intervention?<br>5.2.1.2 What are they saying about the intervention?<br>5.2.1.3 To what extent do they influence others to implement the intervention?	How was the intervention communicated/promoted to leaders/senior managers within your setting/ organization? (OL)<br>To what extent do you think leaders/senior managers influenced others within the organization to implement the intervention? (OL)
5.2.2 Formally Appointed Internal Implementation Leaders (Im)	Individuals from within the organization who have been formally appointed with responsibility for implementing an intervention as coordinator, project manager, team leader, or other similar role.	5.2.2.1 How did your organization/setting/work unit become involved in implementing the intervention?<br>5.2.2.2 How was this decision made? By whom? Were you involved in the process?<br>5.2.2.3 Who leads the implementation of the intervention within your organization/ setting/ work unit? How did they come into this role? (Appointed? Volunteered?)<br>5.2.2.4 What attributes does this person have that makes them effective at implementing?<br>5.2.2.5 Do they have sufficient authority to do what is necessary to implement the intervention?	How did your work unit/organization become involved in implementing the intervention? (Im)<br>Who led/leads the implementation of the intervention in your setting/organization? (Appointed/Volunteered?) (Im)<br>Did the implementer have the required attributes to implement the intervention? (Im)<br>Did the implementer have the sufficient authority to do what was necessary to implement the intervention? (Im)

		5.2.2.6 Who else is involved in leading the implementation of the intervention?	
5.2.3 Champions (Ch)	"Individuals who dedicate themselves to supporting, marketing, and 'driving through' an [implementation]" [101] (p. 182), overcoming indifference or resistance that the intervention may provoke in an organization.	5.2.3.1 Are there other people, apart from the formal implementation leader, who are champions of the intervention (go above and beyond what might be expected)?<br>5.2.3.2 Is this a formal or informal role? What position do they hold within the organization?<br>5.2.3.3 How do they help with implementation of the intervention?<br>5.2.3.4 To what extent do you respect the opinions and actions of these champions?	Apart from the 'implementer' were there other people in your setting/organization who were champions of the intervention? (Ch)<br>What was their contribution to the implementation of the intervention? (Ch)
5.2.4 External Change Agents (CA)	Individuals who are affiliated with an outside entity who formally influence or facilitate intervention decisions in a desirable direction.	5.2.4.1 Is someone (or a team) outside your organization/setting/work unit helping with the implementation of the intervention? Who?<br>5.2.4.2 How did they get involved?<br>5.2.4.3 What is their role?<br>5.2.4.4 What activities will they be doing?<br>5.2.4.5 How helpful do you think they will be? In what ways?	Did you have other people outside of your setting/ organization that helped with implementing the intervention? (CA)<br>What was their contribution to the implementation of the intervention? (CA)
5.2.5 Key Stakeholders (KS)	Individuals who are influential and well-respected	5.2.5.1 Who do you think are the key individuals to get onboard to encourage people to **use** the intervention?<br>5.2.5.2 What steps were/are being taken to encourage key individuals to support the intervention? Who are you targeting? How are you approaching them? What information are you giving them? How are you communicating with them? How frequently?<br>5.2.5.3 What is your communication strategy for getting the word out about the intervention? What materials/modes of communication eg: bulletins, brochures? Processes eg: meetings, informally?	How was the intervention communicated/promoted to key stakeholders outside your setting/organization? (KS)<br>To what extent do you think key stakeholders influenced the target audience to enroll in the intervention? (referrals) (KS)
5.2.6 Intervention Participants (IP)	Individuals who access the intervention.	5.2.6.1 How are you/your colleagues communicating to individuals about the intervention?<br>5.2.6.2 How do/did they participate in the intervention?<br>5.2.6.3 How do/did they access the intervention?	How was the intervention communicated/promoted to the target audience? (IP)

5.3 Executing (Exe)	Carrying out or accomplishing the implementation according to plan.	5.3.1 Was/is the intervention implemented according to the implementation plan? How? Why not?	Was the intervention implemented as per the proposed planned (if there was one)? In what ways? (Exe)
5.4 Reflecting & Evaluating (Eva)	Quantitative and qualitative feedback about the progress and quality of implementation accompanied with regular personal and team debriefing about progress and experience.	5.4.1 What kind of information do/did you collect when implementing the intervention? What measures are being tracked? How are you tracking them? How will this information be used?<br>5.4.2 Will/have you receive feedback reports about the implementation of the intervention? In what format? Frequency? From whom? Compiled by whom? Helpful? Could this be improved?<br>5.4.3 How do/did you assess progress towards implementation or intervention goals? Are results of evaluation distributed to stakeholders?<br>5.4.4 Is/was feedback elicited from staff? What kind of feedback?<br>5.4.5 Is/was feedback elicited from intervention participants? What kind of feedback?<br>5.4.6 To what extent has your organization/ setting/work unit set goals for implementing the intervention? What are these goals? How and to whom are they communicated?	Do you have any comments about the evaluation information that was collected as part of the intervention? (Eva)<br>Did you collect any additional information locally? For what purpose? (Eva)<br>How did you assess if implementation was being effective? Goals were being met? (Eva)<br>Were any feedback reports about the implementation of the intervention compiled/disseminated? Describe (Eva)
